# Greater Body Dissatisfaction at Admission Is Associated With Lower BMI at Discharge in Anorexia Nervosa: Predictive Validity of the Eating Pathology Symptoms Inventory

**DOI:** 10.1002/eat.24560

**Published:** 2025-09-27

**Authors:** Danielle A. N. Chapa, Marianna L. Thomeczek, Brianne N. Richson, Alan Duffy, Kara A. Christensen Pacella, Kelsie T. Forbush, Renee D. Rienecke, Dan V. Blalock, Sara R. Gould, Victoria L. Perko, Philip S. Mehler

**Affiliations:** ^1^ Department of Psychiatry University of Pittsburgh Pittsburgh Pennsylvania USA; ^2^ Department of Psychology University of Kansas Lawrence Kansas USA; ^3^ Sanford Center for Biobehavioral Research Fargo North Dakota USA; ^4^ Department of Psychiatry and Behavioral Science University of North Dakota Fargo North Dakota USA; ^5^ Eating Recovery Center and Pathlight Mood & Anxiety Center Chicago Illinois USA; ^6^ Department of Psychology University of Nevada Las Vegas Las Vegas Nevada USA; ^7^ Department of Clinical Child Psychology University of Kansas Lawrence Kansas USA; ^8^ Lifespan Institute University of Kansas Lawrence Kansas USA; ^9^ Department of Psychiatry and Behavioral Sciences Northwestern University Chicago Illinois USA; ^10^ Department of Psychiatry and Behavioral Sciences Duke University Durham North Carolina USA; ^11^ Center of Innovation to Accelerate Discovery and Practice Transformation (ADAPT) Durham Veterans Affairs Medical Center Durham North Carolina USA; ^12^ Children's Mercy Hospital Kansas City Missouri USA; ^13^ ReefPoint Group Annapolis Maryland USA; ^14^ ACUTE Center for Eating Disorders at Denver Health Denver Colorado USA; ^15^ School of Medicine University of Colorado Denver Colorado USA

**Keywords:** anorexia nervosa, bmi, eating disorders, eating pathology symptoms inventory, predictive validity, psychometrics, treatment outcomes

## Abstract

People with anorexia nervosa (AN) engage in dietary restriction and other weight loss behaviors that result in dangerously low body weight, leading to an increased risk for mortality and medical complications. Weight gain is one of the most important indicators of treatment progress and recovery for AN. There are limited predictors of weight gain for patients with AN, making it difficult for clinicians to anticipate which patients are likely to respond favorably to treatment. Thus, there is a need to identify additional, potentially modifiable predictors of weight gain within a higher level of care for AN. This study tested the predictive validity of the Eating Pathology Symptoms Inventory (EPSI) in adults receiving a higher level of care for AN (*N* = 340). We hypothesized that EPSI scores at treatment admission would predict body mass index (BMI) at discharge. Linear regression was used to identify predictors of discharge BMI. EPSI Body Dissatisfaction at admission (β = −0.043, *p* = 0.005*)* predicted BMI at discharge (controlling for admission BMI, length of stay, and level of care), such that individuals with greater body dissatisfaction at admission had lower BMIs at treatment discharge. Other EPSI scales did not predict BMI. Results supported the predictive validity of EPSI Body Dissatisfaction for discharge weight in adults receiving a higher level of care for AN. Patients who are more dissatisfied with their bodies, despite having a dangerously low BMI at admission, may be at risk for poorer treatment outcomes.

## 1 Introduction

Persons with anorexia nervosa (AN) engage in dietary restriction and other weight loss behaviors that result in dangerously low body weight (American Psychiatric Association 2022; Mason et al. 2018). Weight loss and malnutrition among persons with AN are associated with medical complications, including problems with gastrointestinal, cardiac, pulmonary, neurological, musculoskeletal, and endocrine systems (Leach et al. 2024; Mehler and Brown 2015; Westmoreland et al. 2016). Among all psychiatric illnesses, AN has one of the highest mortality rates due to health complications and completed suicides (Arcelus et al. 2011; Auger et al. 2021; Chesney et al. 2014; Mehler et al. 2022). Given the seriousness of AN, it is critical to understand and quickly identify which patients are at risk for poor treatment outcomes. Information about anticipated prognosis could shape clinicians' initial decisions about the level of care and treatment intensity.

Although recovery definitions vary (Couturier and Lock 2006), weight restoration is one important indicator of recovery from AN (Bardone‐Cone et al. 2010; Couturier and Lock 2006) and the primary target of most treatment programs. The best available treatments for AN lead to significant end‐of‐treatment weight gain; however, many patients do not attain full weight restoration, and others are unable to maintain weight recovery post‐discharge (Kaplan et al. 2009; Murray et al. 2019). Individuals who do not achieve full weight restoration or do not maintain discharge weight often require multiple hospitalizations, referred to as the “revolving door” phenomenon (Marzola et al. 2021). Incomplete recovery and/or recurrent admissions are problematic because of the physical and psychological risks associated with AN and the high cost of eating‐disorder treatment (Stuhldreher et al. 2012).

Greater weight gain and faster weight gain during treatment are predictors of improved outcomes for AN at discharge and short‐term follow‐up (Kaplan et al. 2009; Lock et al. 2013; Lund et al. 2009). For example, in one study, greater weight gain within the first two weeks of inpatient treatment predicted higher weight at discharge, as well as a higher likelihood of having a discharge weight within the normal range (Kolar et al. 2022). Faster rates of weight gain during treatment predicted a larger increase in body mass index (BMI) from baseline to 12‐month follow‐up (Cartwright et al. 2017), a higher likelihood of weight restoration at discharge (Makhzoumi et al. 2017), and more weight maintenance at 6‐month follow‐up (Makhzoumi et al. 2017). Although early and rapid weight gain predicts AN treatment outcomes, there is a need to better understand treatment response predictors that can be measured prior to treatment. Weight suppression (i.e., the discrepancy between one's highest ever weight for their height and one's lowest ever weight for their height) and BMI at treatment initiation are routinely assessed predictors of post‐treatment weight outcomes for AN (Berner et al. 2013; Sly and Bamford 2011), and admitting BMI predicts length of treatment (Kan et al. 2021; Kästner et al. 2018). Although admission weight and weight history can inform clinical decisions on the appropriate level of care, there is a need to identify additional, potentially modifiable predictors of discharge BMI to further inform the level of care for a given patient and provide additional intervention or support, as needed.

Self‐report questionnaires are often completed upon treatment intake, and thus would ideally provide an early index of possible treatment trajectory. The Eating Disorder Examination—Questionnaire ([EDE‐Q]; Fairburn 2008) is one of the most commonly used measures to monitor eating‐disorder psychopathology (Reilly et al. 2025), despite inconsistent evidence that the EDE‐Q predicts outcomes of interest for AN. For example, EDE‐Q subscales predicted treatment outcomes and rates of weight gain in the context of outpatient cognitive behavioral therapy (Lockwood et al. 2012; Marcoulides and Waller 2012; Ricca et al. 2010), but not in a specialized inpatient treatment for AN (Wales et al. 2016). Mixed findings may result from differences in levels of care (e.g., higher levels of care consist of increased oversight and control of the therapeutic environment and serve individuals with more severe presentations), treatment modality, statistical approach (e.g., controlling for age of onset, AN subtype, sex, and other comorbidities), and the inconsistent factor structure of the EDE‐Q (Berg et al. 2012; Jenkins and Rienecke 2022; Jenkins et al. 2024). Given the mixed evidence as to whether the EDE‐Q is predictive of weight status at the end of treatment, further research that incorporates additional measurement tools is needed. Examining other self‐report measures that might provide greater predictive validity could increase the ability to accurately predict prognosis and effectively triage care.

One such tool is the Eating Pathology Symptoms Inventory ([EPSI]; Forbush et al. 2013). The EPSI has many strengths, including evidence of a consistent factor structure, internal consistency reliability, and construct validity (Forbush et al. 2014, 2013). The EPSI has also exhibited strong replication of its factor structure and measurement invariance across a variety of demographic groups, including adolescents (Richson et al. 2021), men (Forbush et al. 2013), transgender and gender‐diverse adults (Nagata, Otmar, Lopez, et al. 2025), and sexual minority adults (Nagata, Otmar, Kim, et al. 2025; Perko et al. 2021), that support its widespread use in clinical settings. Although the EPSI has shown evidence for criterion validity in predicting eating‐disorder treatment recovery when combined with a measure of depression and anxiety (Forbush et al. 2018, 2024), no past studies have evaluated the EPSI's independent predictive validity for BMI at discharge from intensive treatment among persons with AN. Thus, the purpose of the current study was to test whether admission scores on the EPSI predicted BMI at discharge among adults with AN. We hypothesized that EPSI scales at admission would prospectively predict BMI at the end of treatment, with EPSI Restricting scale scores emerging as a strong indicator of discharge BMI.

## 2 Method

### 2.1 Participants and Procedures

These data were collected at Eating Recovery Center (ERC) sites across the United States between 2016 and April 2020. ERC is a large multisite eating‐disorder treatment facility offering inpatient (IP), residential (RES), partial hospitalization (PHP), and intensive outpatient (IOP) care. Patients were admitted to, and progressed through, the appropriate level(s) of care based on medical stability and behavioral progress (e.g., stepping up to a higher level of care or stepping down to a lower level of care, as needed). Treatment at the IP and RES levels of care was 24/7 onsite. PHP treatment was 7 days per week for 8–10 h per day, and patients returned home or to a non‐treatment residence each night. IP, RES, and PHP included twice weekly individual therapy, weekly family therapy, twice weekly medical provider/psychiatric visits (daily for IP), weekly registered dietitian meetings, and 3–4 h of evidence‐based skills groups per day. Patients ate three supervised meals and 2–3 supervised snacks daily. Patients in IOP care received three, 3‐h group psychoeducation sessions weekly, 1 h of individual or family therapy weekly, dietary appointments every other week, and medical monitoring weekly.

This study was approved by the Salus Institutional Review Board. Diagnostic interviews were completed prior to admission. Patients completed intake paperwork at the time of admission, which included consent for their medical chart information to be used for research purposes, as well as self‐reports of eating‐disorder psychopathology. At the end of treatment, which varied in length, each patient was asked to complete discharge assessments, including repeated height and weight measurements and several self‐report assessments.

Participants were adults with AN (*N* = 532) who were admitted for their first course of treatment at the ERC between 2016 and April 2020 and completed the EPSI at admission. Individuals met criteria for either AN restricting or binge purge subtype. Individuals with an admission BMI greater than 19.5 kg/m^2^ (*n* = 117) or individuals who were missing discharge BMI in the research database (*n* = 75) were excluded from the current study analyses. The subset of individuals with a BMI greater than 19.5 kg/m^2^ was a heterogeneous group, including individuals with atypical AN, AN in partial remission, or sub‐threshold AN and were therefore excluded to minimize statistical “noise” in the data. Thus, the final analytic sample included 340 adults with AN. A BMI of 19.5 was chosen because it would include individuals who did not meet a strict BMI cut‐off but still had a BMI that was close to underweight and thus warranted weight restoration.

## 3 Measures

### 3.1 Eating Pathology Symptoms Inventory (EPSI)

The EPSI (Forbush et al. 2013) is a 45‐item self‐report questionnaire used to assess eating‐disorder psychopathology over the past four weeks. The EPSI is comprised of eight scales which include Body Dissatisfaction, Binge Eating, Cognitive Restraint, Purging, Restricting, Excessive Exercise, Negative Attitudes toward Obesity, and Muscle Building (Forbush et al. 2013). Participants responded on a five‐point Likert scale ranging from “0 = *Never*” to “4 = *Very Often*,” and items were summed to yield a total score for each scale. The eight‐factor structure of the EPSI has been replicated in clinical and non‐clinical samples (Coniglio et al. 2018; Forbush et al. 2013, 2014). The EPSI demonstrated evidence for excellent internal consistency among eating disorder patients receiving a higher level of care (Forbush et al. 2013; Joiner et al. 2022; Reilly et al. 2021). Scores on the EPSI scales at admission were used for the present analysis. Internal consistency reliability for EPSI scales was good to excellent with Cronbach's alpha values that ranged from 0.73 for Muscle Building to 0.95 for Excessive Exercise.

### 3.2 Body Mass Index (BMI)

Admission and discharge BMI (kg/m^2^) was calculated using measured height and weight at admission or weight at discharge.

## 4 Statistical Analyses

All analyses were completed in R Version 4.4.1 (R Core Team 2024) using base functions and the *corrplot* package (van der Laken 2023). First, descriptive statistics were used to characterize sample demographic and clinical characteristics. Means and standard deviations were calculated for all continuous and ordinal variables (age, admission BMI, discharge BMI, length of stay, EPSI scale scores at admission), and frequency tabulations were calculated for all nominal variables (sex, gender, race, ethnicity, AN subtype). Second, Pearson *r* correlations were estimated to evaluate possible multicollinearity among linear regression predictor variables. Third, the full linear regression model (that included all EPSI scales to predict Discharge BMI with Admission BMI as a covariate) was evaluated after checking model assumptions (e.g., residual vs. fitted plot, QQ plot, Shapiro–Wilk test) and evaluating the variable inflation factor (VIF). No correlation between predictor variables is present when VIF = 1. VIF values > 1 and < 5 suggest moderate correlations between predictor values, whereas VIF values ≥ 5 suggest high correlations (Shrestha 2020). No variable transformations were required, and there were no missing data for the independent or dependent variables. Fourth, one covariate was added at a time using a hierarchical regression approach (length of stay, level of care, AN subtype, age, sex, treatment site). Bonferroni's correction was applied for the number of EPSI scales evaluated (i.e., 0.05/8), and a *p*‐value of 0.006 was used.

## 5 Results

The mean (SD) age of patients at treatment admission was 26.5 (9.2) years, and ages ranged from 18 to 63 years. BMI values at admission ranged from 11.9 to 19.5 kg/m^2^; the mean (SD) BMI was 16.6 (1.6) kg/m^2^. Most patients self‐identified as women, White, and Non‐Hispanic. See Table 1 for additional demographic and clinical characteristics. Mean scores on the EPSI at admission in this sample were comparable to norms for patients with a diagnosis of AN (Forbush et al. 2013). See Table 2 for correlations among EPSI scales at admission, BMI at admission, BMI at discharge, and length of stay.

**TABLE 1:** Demographic and clinical characteristics.

Variable	*n* (*%*)/*M* (SD)
Gender	
Women	313 (92.1%)
Men	25 (7.4%)
Transgender	1 (0.3%)
Prefer not to answer	1 (0.3%)
Race/ethnicity	
Asian	13 (3.8%)
Black	2 (0.6%)
Hispanic/Latino	9 (2.6%)
White	285 (83.8%)
Multiracial	11 (3.2%)
Prefer not to answer	5 (1.5%)
Missing	15 (4.4%)
Anorexia nervosa subtype	
Restricting	184 (54.1%)
Binge/purge	156 (45.9%)
Admission level of care	
Inpatient/residential	199 (58.5%)
Intensive outpatient/partial hospitalization	35 (10.3%)
Missing	106 (31.2%)
Age at admission (years)	26.5 (9.2)
Admission BMI (kg/m^2^)	16.6 (1.6)
Discharge BMI (kg/m^2^)	19.8 (1.7)
Length of stay (days)	67.9 (37.4)
EPSI scale scores at admission	
Binge eating	8.14 (7.71)
Body dissatisfaction	18.04 (7.58)
Cognitive restraint	8.60 (3.49)
Excessive exercise	9.04 (6.91)
Muscle building	2.97 (3.69)
Negative attitudes toward obesity	8.76 (6.04)
Purging	5.47 (5.94)
Restricting	15.84 (5.78)

Abbreviation: BMI = body mass index.

**TABLE 2:** Correlation matrix of admission eating pathology symptom inventory scale scores, body mass index, and length of stay.

	BE	BD	CR	MB	P	EE	NATO	R	Adm_BMI	Dis_BMI	LOS
BE	1	0.003	−0.164 **	0.196***	**0.234** ***	−0.114*	0.105	**−0.244** ***	0.080	−0.021	−0.126*
BD	0.003	1	**0.588** ***	−0.103	**0.309** ***	**0.285** ***	**0.298** ***	**0.536** ***	0.111*	−0.021	0.020
CR	−0.164**	**0.588** ***	1	−0.001	**0.224** ***	**0.514** ***	0.196***	**0.483** ***	0.030	0.083	0.102
MB	0.196***	−0.103	−0.001	1	**0.231** ***	0.153**	0.179***	−0.084	0.055	0.044	0.015
P	**0.234** ***	**0.309** ***	**0.224** ***	**0.231** ***	1	0.134*	**0.265** ***	**0.248** ***	**0.208** ***	0.100	0.015
EE	−0.114*	**0.285** ***	**0.514** ***	0.153**	0.134*	1	0.165**	**0.273** ***	0.050	0.073	0.080
NATO	0.105	**0.298** ***	0.196***	0.179***	**0.265** ***	0.165**	1	**0.222** ***	0.144*	0.052	−0.102
R	**−0.244** ***	**0.536** ***	**0.483** ***	−0.084	**0.248** ***	**0.273** ***	**0.222** ***	1	0.085	0.073	0.005
Adm_BMI	0.080	0.111*	0.030	0.055	**0.208** ***	0.050	0.144*	0.085	1	**0.499** ***	−0.045
Dis_BMI	−0.021	−0.021	0.083	0.044	0.100	0.073	0.052	0.073	**0.499** ***	1	**0.435** ***
LOS	−0.126*	0.020	0.102	0.015	0.015	0.080	−0.102	0.005	−0.045	**0.435** ***	1

*Note*: Bolded values represent correlations > |0.20|.

Abbreviations: Adm_BMI = admission body mass index; BD = body dissatisfaction; BE = binge eating; CR = cognitive restraint; Dis_BMI = discharge body mass index; EE = excessive exercise; EPSI = eating pathology symptoms inventory; LOS = length of stay; MB = muscle building; NATO = negative attitudes toward obesity; *P* = purging; *R* = restricting.

* *p* < 0.05.

** *p* < 0.01.

*** *p* < 0.001.

### 5.1 Model 1: Full EPSI

The linear regression model that included all EPSI scales at admission with admission BMI as the sole covariate predicted significant BMI at discharge, *F*(9, 326) = 15.15, *p* < 0.001 (Adjusted R^2^ = 0.276). Only Body Dissatisfaction at admission emerged as a significant a predictor of BMI at discharge. Greater EPSI Body Dissatisfaction scores at admission predicted lower BMI at discharge (*B* = −0.045, *p* = 0.003) (Table 3). VIF values ranged from 1.20 for EPSI Muscle Building to 2.01 for EPSI Cognitive Restraint, indicating that high levels of multicollinearity were not present in the model.

**TABLE 3:** Unstandardized and standardized regression coefficient estimates.

Variable	Unstandardized beta	Standardized beta	Standard error	T statistic	Partial *R* ^2^
Model 1: full model, admission BMI covariate					
Binge eating	−0.005	−0.023	0.012	−0.450	0.001
**Body dissatisfaction**	**−0.045**	**−0.196**	**0.015**	−3.012**	**0.027**
Cognitive restraint	0.080	0.161	0.033	2.429*	0.018
Excessive exercise	0.003	0.011	0.014	0.204	< 0.001
Muscle building	0.005	0.011	0.024	0.214	< 0.001
Negative attitudes toward obesity	−0.0004	−0.001	0.015	−0.028	< 0.001
Purging	0.001	0.005	0.016	0.095	< 0.001
Restricting	0.014	0.046	0.018	0.776	0.002
Admission BMI	0.557	0.528	0.051	11.015***	0.271
Model 2: full model, admission BMI and length of stay covariates					
Binge eating	0.007	0.034	0.011	0.661	0.002
**Body dissatisfaction**	**−0.041**	**−0.184**	**0.014**	−2.901**	**0.031**
Cognitive restraint	0.044	0.092	0.031	1.425	0.008
Excessive exercise	0.009	0.038	0.013	0.695	0.002
Muscle building	0.008	0.016	0.025	0.324	< 0.001
Negative attitudes toward obesity	0.005	0.018	0.014	0.351	< 0.001
Purging	−0.003	−0.011	0.016	−0.195	< 0.001
Restricting	0.029	0.100	0.017	1.698	0.011
Admission BMI	0.517	0.484	0.050	10.255***	0.287
Length of stay	0.021	0.454	0.002	9.668***	0.264
Model 3: full model, admission BMI, length of stay, and level of care covariates					
Binge eating	−0.001	−0.002	0.012	−0.044	< 0.001
**Body dissatisfaction**	**−0.043**	**−0.188**	**0.015**	−2.832**	**0.036**
Cognitive restraint	0.053	0.108	0.033	1.593	0.012
Excessive exercise	0.005	0.018	0.014	0.326	< 0.001
Muscle building	−0.002	−0.004	0.027	−0.089	< 0.001
Negative attitudes toward obesity	0.015	0.051	0.015	0.966	0.004
Purging	−0.006	−0.021	0.017	−0.372	0.001
Restricting	0.013	0.044	0.018	0.727	0.002
Admission BMI	0.664	0.581	0.060	11.089***	0.365
Length of stay	0.023	0.497	0.002	10.230***	0.328
Level of care	−0.945	−0.192	0.259	−3.650***	0.059

*Note*: Bolded rows indicate significant predictive variables. The model included all EPSI scales without controlling for covariates. Models 2–4 used hierarchical regression to add one covariate at a time. To evaluate possible effects of multicollinearity, the EPSI Body Dissatisfaction and Cognitive Restraint scales were entered independently (Models 5–6). The other six EPSI scales were also entered independently in isolation, and they did not predict BMI. *p < 0.05; **p < 0.01; ***p < 0.001.

Abbreviation: BMI = body mass index.

### 5.2 Models 2–3: Hierarchical Regression‐Full EPSI With Additional Covariates

Results of Model 2, which included all EPSI scales and admission BMI and length of stay as covariates, significantly predicted BMI at discharge, *F*(10, 261) = 21.27, *p <* 0.001 (Adjusted *R* ^2^ = 0.428). Similar to Model 1, Body Dissatisfaction (*B* = −0.041, *p* = 0.004) predicted BMI at discharge. Length of stay was a significant covariate (*p* < 0.001); individuals with a longer admission had a higher BMI at discharge. Results of Model 3, which included all EPSI scales and three covariates (admission BMI, length of stay, and level of care), also predicted significant BMI at discharge, *F*(11, 214) = 21.67, *p <* 0.001 (Adjusted R^2^ = 0.503). EPSI Body Dissatisfaction remained a robust predictor; greater EPSI Body Dissatisfaction scores at admission predicted lower BMI at discharge (B = −0.043, *p* = 0.005), holding other EPSI scale scores constant and controlling for admission BMI, length of stay, and level of care (Table 3). Results of Models 4–7, which added one additional covariate at a time in addition to admission BMI, length of stay, and level of care (e.g., AN subtype, age at admission, sex, and treatment site), indicated that additional covariates were not significant predictors of discharge BMI and were therefore not included in the model results (Table 3).

## 6 Discussion

The current study aimed to test whether admission scores on the EPSI predicted BMI at discharge among adults with AN. We hypothesized that EPSI scales would predict discharge BMI, with the Restricting scale predicting the largest proportion of the variance. Inconsistent with our hypotheses, the Restricting scale was not a significant predictor of discharge BMI. In partial support of our hypothesis, one EPSI scale, Body Dissatisfaction, emerged as a robust predictor of BMI at discharge. Overall, these results suggest that psychological factors at admission may be more predictive of BMI at discharge than behavioral factors. Greater levels of body dissatisfaction at treatment admission predicted lower BMI at discharge, and this finding was robust to the addition of model covariates (i.e., length of stay, level of care). Findings were consistent with previous research, which identified pre‐treatment levels of EDE‐Q Shape and Weight Concerns as significant predictors of end‐of‐treatment outcomes for individuals with AN receiving outpatient CBT (Ricca et al. 2010). Body dissatisfaction and overvaluation of weight and shape are highly related, overlapping constructs, as evidenced by high positive correlations in patients with an eating disorder across higher (*r*'s = 0.66–0.72; Forbush et al. 2013) and lower (*r*'s = 0.84–0.89; Coniglio et al. 2018) levels of care.

The current study's finding that greater body dissatisfaction at intake was associated with lower BMI at the end of intensive treatment points to the potential of body dissatisfaction as an important indicator of treatment prognosis and eating‐disorder severity. The current study did not evaluate if temporal changes in body dissatisfaction predicted later changes in BMI. Future studies that are designed to tease apart the role of body dissatisfaction in eating disorder recovery are imperative. Understanding and intervening on factors that predict weight gain is important because reaching a discharge BMI of ≥ 19 has been found to predict maintaining a BMI ≥ 19 at a 6‐month follow‐up for patients who were hospitalized for AN (Redgrave et al. 2021). Body dissatisfaction is generally not intervened on until later in treatment (i.e., following substantial weight and cognitive recovery). For some patients, providers in higher levels of care may consider treating body dissatisfaction earlier. Furthermore, our findings support previous research that body dissatisfaction may predict future outcomes; for example, in one study, body dissatisfaction in adolescence predicted disordered eating 10 years later (Foster et al. 2024). It is possible that elevated and/or unresolved body dissatisfaction following discharge could place someone at risk for poorer treatment outcomes as well as future relapse.

Inconsistent with our hypotheses, restricting behaviors at admission were not indicative of discharge BMI among individuals with AN. There are several reasons why purposeful restriction of food intake in the month leading up to admission did not predict BMI. First, this finding may have emerged due to a restriction of range as most participants reported high levels of restriction in the current study, which is consistent with a diagnosis of AN. Second, the severity of restrictive behaviors may have already been sufficiently captured by admission BMI and length of stay covariates. Finally, it could be that dietary restriction levels are not directly related to BMI outcomes in a higher level of care where there are limited opportunities to engage in dietary restriction. Levels of dietary restriction may be more relevant for outcomes in a lower level of care (e.g., outpatient) where individuals have more autonomy over their eating.

There are several limitations of the current study that are important to note. First, the current sample was comprised of primarily White, cisgender women able to access higher levels of care, which limits the generalizability to more diverse and/or disadvantaged samples. Many individuals from diverse backgrounds may experience more barriers to eating‐disorder treatment (Cachelin et al. 2001), potentially setting them up for poorer outcomes. Additional research is needed to test whether results replicate in samples comprised of individuals with different racial, ethnic, and gender identities. Second, the sample included individuals receiving treatment across higher levels of care, and treatment approaches may have varied slightly from site to site. However, this type of variation in care may be representative of treatment received in the ‘real world’ and may increase the generalizability of results, particularly given that findings were not altered by adding site as a covariate. It is also important to note that adding the level of care as a covariate did not change the main findings. Third, BMI is only one important outcome of interest (Bardone‐Cone et al. 2018), and the present study did not speak to the predictive validity of the EPSI for other aspects of AN outcomes and recovery (e.g., improvement in eating‐disorder severity, clinical impairment, well‐being, medical complications) or established indicators of prognosis (e.g., rate of weight gain). The current study functionally accounted for the rate of weight change by controlling for length of stay, and future research should systematically examine if baseline scores on the EPSI and other self‐reported measures of ED psychopathology predict early weight gain in treatment for AN. Fourth, these findings cannot necessarily be extended to individuals with atypical AN, who may present with relatively higher body dissatisfaction and eating‐disorder psychopathology (see review/meta‐analysis: Johnson‐Munguia et al. 2024). Finally, the EPSI scales explained a small amount of variance in discharge BMI. Of note, EPSI Body Dissatisfaction explained additional variance in discharge BMI above and beyond Admission BMI. Additional studies replicating the role of the EPSI Body Dissatisfaction on AN outcomes are necessary.

There were also several important strengths of the current study. First, to our knowledge, this was the first study to investigate the EPSI's predictive validity among individuals seeking treatment for AN. Second, the sample was inclusive of a relatively even split of individuals with AN restricting and binge‐purge subtypes, which supported the predictive validity of the EPSI for both AN presentations. Finally, the current study was conducted using data from a large multisite treatment center with locations in various regions of the United States, which may highlight the predictive validity of the EPSI in ‘real world’ treatment settings. Future research is needed to examine the validity of the EPSI for predicting BMI outcomes and other relevant indicators of recovery (e.g., discharge BMI relative to target goal weight, presence of eating disorder cognitions, presence of other eating disorder behaviors) in more diverse samples, among adolescent patients with AN, among patients with atypical AN, and in outpatient treatment settings. Additional research evaluating the incremental validity of the EPSI relative to other self‐reports of eating disorder psychopathology would help inform assessment battery selections, particularly as it relates to informing treatment prognosis. Studies investigating how change in EPSI scores during treatment relate to eating‐disorder symptom change would inform how interventions can be refined to maximally target symptomatology most predictive of lasting health and recovery.

In conclusion, greater EPSI Body Dissatisfaction at treatment admission for AN, a time when individuals are already at a dangerously low BMI, predicted lower BMI at discharge. Assessing body dissatisfaction as a prognostic tool via the EPSI may be helpful when treatment planning at higher levels of care for patients with AN in order to mitigate the potential for poor outcomes.

## Author Contributions

**Danielle A. N. Chapa:** conceptualization, formal analysis, writing – original draft, writing – review and editing. **Marianna L. Thomeczek:** data curation, writing – review and editing. **Brianne N. Richson:** data curation, writing – review and editing. **Alan Duffy:** data curation, writing – original draft, writing – review and editing. **Kara A. Christensen Pacella:** writing – review and editing. **Renee D. Rienecke:** conceptualization, writing – review and editing, writing – original draft. **Dan V. Blalock:** formal analysis, writing – review and editing. **Sara R. Gould:** conceptualization, writing – review and editing. **Victoria L. Perko:** writing – original draft. **Philip S. Mehler:** writing – review and editing. **Kelsie T. Forbush:** conceptualization, writing – review and editing.

## Conflicts of Interest

Dr. Blalock receives consulting fees from Eating Recovery Center/Pathlight Mood and Anxiety Center. Dr. Renee Rienecke receives consulting fees from the Training Institute for Child and Adolescent Eating Disorders LLC, and receives royalties from Routledge and Hachette Book Group Inc.

## Data Availability

The data that support the findings of this study are available on request from the corresponding author. The data are not publicly available due to privacy or ethical restrictions.
